# Driver Drowsiness Multi-Method Detection for Vehicles with Autonomous Driving Functions

**DOI:** 10.3390/s24051541

**Published:** 2024-02-28

**Authors:** Horia Beles, Tiberiu Vesselenyi, Alexandru Rus, Tudor Mitran, Florin Bogdan Scurt, Bogdan Adrian Tolea

**Affiliations:** Department of Mechanical Engineering and Automotive, University of Oradea, Universitatii St. 1, 410087 Oradea, Romania; horia.beles@uoradea.ro (H.B.); vtiberiu@uoradea.ro (T.V.); alrus@uoradea.ro (A.R.); scurt.florinbogdan@didactic.uoradea.ro (F.B.S.); bogdan.tolea@uoradea.ro (B.A.T.)

**Keywords:** autonomous driving, ADAS, sensor integration, sensors fusion, deep learning, video signal processing

## Abstract

The article outlines various approaches to developing a fuzzy decision algorithm designed for monitoring and issuing warnings about driver drowsiness. This algorithm is based on analyzing EOG (electrooculography) signals and eye state images with the aim of preventing accidents. The drowsiness warning system comprises key components that learn about, analyze and make decisions regarding the driver’s alertness status. The outcomes of this analysis can then trigger warnings if the driver is identified as being in a drowsy state. Driver drowsiness is characterized by a gradual decline in attention to the road and traffic, diminishing driving skills and an increase in reaction time, all contributing to a higher risk of accidents. In cases where the driver does not respond to the warnings, the ADAS (advanced driver assistance systems) system should intervene, assuming control of the vehicle’s commands.

## 1. Introduction

Driving in a drowsy state is a major problem worldwide. According to a CDC study (Centers for Disease Control and Prevention) from the United States [1], an estimated 1 in 25 drivers older than 18 report falling asleep while driving in the last 30 days. The NHTSA (National Highway Traffic Safety Administration) estimates that in 2017, drowsy driving was responsible for 91,000 crashes. Due to the high number of crashes, there were a resulting 50,000 injuries and nearly 800 deaths [2,3]. According to the EU Mobility and Transport Road Safety Commission, the causes of 10 to 25% of all road accidents in Europe are due to the drowsy state of the driver [4]. It has been observed that the frequent situation in which fatigue affects the driver’s attention is when they are driving on the highway or on roads between cities, due to the monotony of driving activities.

It is well known that technology has advanced at a rapid pace, making it possible to use it in various driving activities. The role of the most functions implemented in vehicles is to reduce these high numbers of accidents and fatalities by two main methods: by warning the driver of possible dangers and/or by direct intervention in the control of the vehicle (automated/autonomous driving).

**Figure 1:**
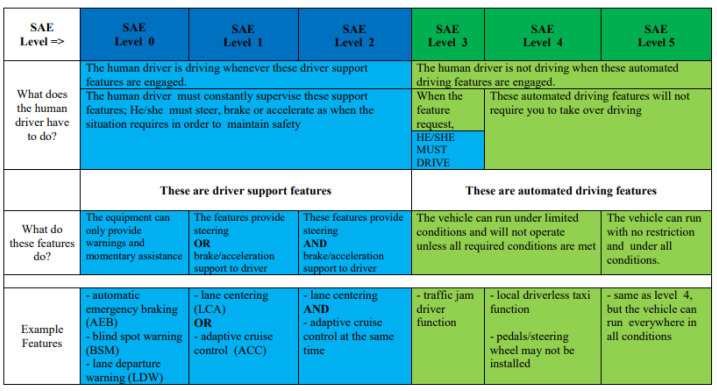
SAE J3016 standard for levels of driving automation [5].

Regarding the automation of driving functions, according SAE International (Society of Automotive Engineers) standards—the J3016 standard [5]—there are 6 levels, from Level 0 (no automation) to Level 5 (fully autonomous/automated vehicle). In order to take into account the differences in automation and to make a clearer distinction between the SAE levels shown in Figure 1, all 6 levels will be briefly explained in the following paragraphs.

SAE Level 0: the systems installed on these vehicles are mostly for warning, not for intervention, so the level has no driving automation functions.

SAE Level 1: the system could take over the control of the acceleration OR brake OR the steering wheel (in order to maintain the direction of travel). The systems present in these vehicles could be the following: adaptive cruise control or lane centering. The driver must be capable of intervening if the traffic situation so requires, because the systems are only for driver assistance.

SAE Level 2: the system is able to control steering, acceleration AND braking, when the functions are active (lane centering and adaptive cruise control working together). At this level (partial automation or assistance) the driver should still monitor the environment and be prepared to intervene and take control of the vehicle when the situation calls for it.

SAE Level 3: the vehicle can monitor the surrounding area and control the steering wheel, brakes and acceleration in some cases of travel. At this level it is conditional automation, and the driver still must be able to take over the control if the situation requires that.

SAE Level 4: also known as ‘high automation’, this level is represented by an autonomous driving system that takes over the driving and monitoring activities during the movement (functions like a local driverless taxi). At this level, the pedals and/or steering wheel may or may not be present.

SAE Level 5 (full automation): this level consists of a full automation system which permits the vehicle to manage all the situations for safe driving in any conditions of traffic.

Based on the SAE International classification, it can be seen that up to level 3 of autonomy, the human driver cannot be eliminated, even if sometimes they are only responsible for monitoring the autonomous driving system, or intervening when it is required. For this reason, driver drowsiness state in a Level 3 or lower autonomous vehicle can be extremely dangerous and need to be monitored.

Detecting driver fatigue can be carried out in the early stages of drowsiness and the driver can be warned. Over time, several methods have been proposed to determine driver fatigue: analysis of the driver’s movements and face expressions, or biological signals measures that relate to monitoring of the driver behavior and vehicle movement [6]. The method of analyzing the driver’s facial movements and expressions includes techniques for measuring mouth movements, eye closing, eye blinking, and head position [7,8,9,10].

The methods based on biological measures that relate to driver bio-signals includes physiological signals such as electrooculography (EOG), electromyogram (EMG), electroencephalogram (EEG), and electrocardiogram (ECG) [11,12,13,14,15,16,17,18,19].

The methods based on behavioral monitoring and vehicle motion include driving behavior, such as time in lane crossing, speed, steering wheel angle, lane position, etc. [7,18]. Some of these methods and other have been studied in detail, and moreover the advantages and disadvantages of each have been addressed in other articles. Combining several methods in a hybrid system would lead to an efficient drowsiness detection system.

In our previous studies, a system for fatigue detection by acquiring and processing encephalogram (EEG) signals was developed, and it is presented in [9,20]. In early research [8], the possibilities of acquiring electromyogram (EMG) signals by three sensors are addressed, and artificial neural networks were used to classify driver fatigue states [9,20]. In [9,20], we presented the driver fatigue state detection system’s performance in the analysis of the driver’s eye position (open, closed, or half-open) based on images acquired while driving.

This paper presents the experiments performed by the authors in order to study the applicability of the integration of different biological parameters of a driver in a multi-sensor system for drowsiness detection. The paper presents a system which could use two signals obtained from biological sensors: EEG and EOG, and two kinds of images: driver face images and eye images. Images of the driver’s face were used to distinguish between face images with closed and open eyes. EEG and EOG signals were acquired in laboratory settings, while driver face images were acquired during driving. In addition to the previous research, this article also presents some suggestions for the implementation of this driver drowsiness monitoring system in vehicles with ADAS.

## 2. Materials and Methods

Devices used for research: NI usb 6003 acquisition board, EMG MIKROE-2623 sensors, Matlab R2022b software version 9.13 and Blackbox L300-1 video camera.

### 2.1. Drowsiness Detection System Based on EOG Signals and Face Image Analysis

**Figure 2:**
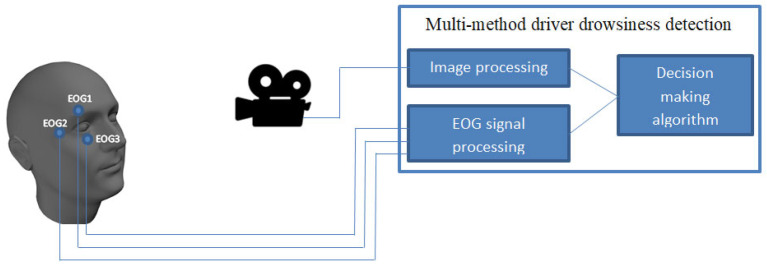
Schematics of a multi-method driver drowsiness detection system based on EOG signals and face image analysis [9].

The main methods which are used in this case are those based on electrooculography (EOG) signals and image classification of the eyelid position (closed or open). For the eye movements, we used an EOG tracking method by measuring eye muscle signals. The state of the eye (closed or open) is monitored through eye image processing. The development of such a system was discussed by some of the authors in [8]. The system presented in [8] is shown in Figure 2. The methods used in the system presented in previous work has its advantages but also some disadvantages. For example, EEG (especially) and EOG sensors presents a major discomfort due to the fact they have to be fixed with a conductive gel, and in some cases, devices must transmit the signal by wire (for a better data transfer). Important research in advanced materials and MEMS (microelectromechanical system) technology is solving some of the problems using some kind of dry electrodes for EEG, presented in [21], or other types of electrodes used, such as, for example, in fitness bracelets. The main developments and findings in this field are encouraged by efforts and the necessity of creating brain–computer interfaces [22,23,24] for various applications, including systems that could allow doctors to monitor patients or help people with disabilities.

### 2.2. System for Drowsiness Detection Based on Face Image Analysis and the Eye Aspect Ratio Algorithm (EAR)

**Figure 3:**
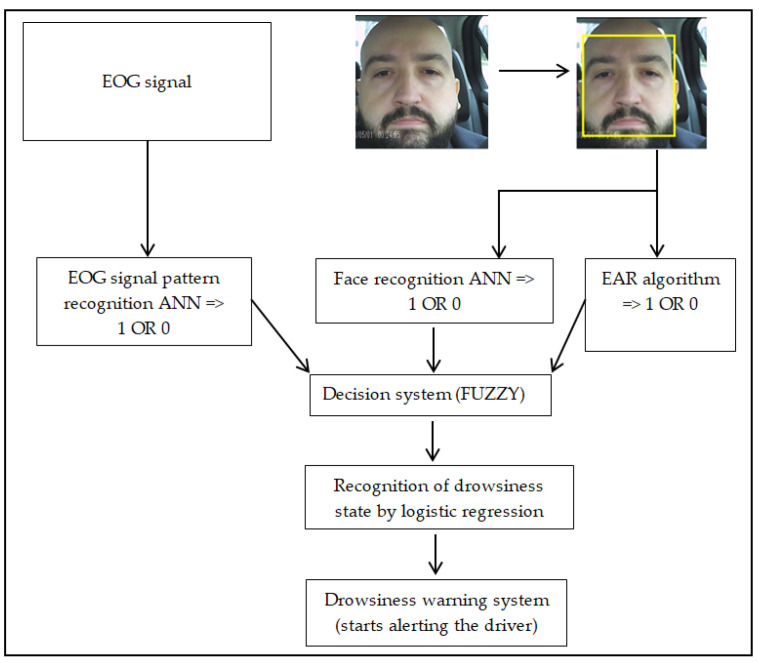
Schematics of a drowsiness detection system based on EEG signals; face image analysis and the EAR algorithm.

In the case of this drowsiness detection system the driver’s face image is used as input. The images are collected during the driving period and streamed to be analyzed [20,25]. After the camera captures the human driver’s images, the images have to be processed by a face/eye recognition algorithm. After this, they are processed by two different variants (of algorithms): the first is an artificial neural network (ANN) which is trained to recognize the position of the driver’s eyelids (open or closed), and the second variant is to use an algorithm for the eye aspect ratio (EAR), which can also provide data about the state of the eyelids (open/closed), but based on a different process. The final step in the image processing process, based on using a trained ANN to recognize a certain driver behavior, is the decision regarding whether the driver is tired and whether or not a warning is needed. For the decision, we used a well-known logistic regression, which is widely used in various fields (including medicine, finance, and machine learning) for tasks like prediction, and it has only two possible outcomes (usually 0 and 1, or ‘yes’ and ‘no’). At this point, the frequency and distribution of blinks can be analyzed to determine if the driver is sleepy or in an alert state. The system’s scheme is shown in Figure 3.

In the following paragraphs, the processing methods and the results obtained after testing and training are represented. MATLAB R2022b tools were used for the image (face/eyes) recognition through an ANN algorithm and also in order to develop the fuzzy decision algorithm [20,26,27]. To train the neural networks, more than 800 min were recorded while the vehicle was driving on public roads (municipal roads, expressways, and motorways), at different stages of driver fatigue and in different weather conditions, but ultimately, relevant sequences were extracted from this large amount of collected data. Thus, several 60-s video sequences (of 1975 frames each) recorded while driving were used to train and test the face image analysis system [20]. Information on driver monitoring in traffic is presented in Table 1.

Column one of the table shows the number of the day and the route on which the record was made. Column two shows how long the driver slept before driving. The third column shows the route followed by the driver; the route can be viewed using the GoogleMaps application, as a link is provided for each route. The fourth column shows how many minutes were recorded for each route. The fifth column describes the weather conditions under which the tests were carried out. The penultimate column describes the time intervals in which the recordings were made. The driver tried to follow his usual daily routine while including the 4 trips shown in the table. All trips were made without breaks for the driver. In addition to video monitoring, the driver was accompanied and supervised by a passenger, who tried to note as many signs of fatigue and inattention as possible during the journey. Thus, the last column in the table shows a summary of the passenger’s notes. The first signs of fatigue started to appear only after about an hour of driving. Another quite important aspect noticed was that on the stretches of road where driving was monotonous (motorway—trip 1; road in alignment—trip 2, 3, 4; or road known by the driver), fatigue states appeared frequently. In addition to the monotonous driving (without much driving activity) which induced a feeling of fatigue/boredom, the driver’s attention to the road decreased. This was especially evident on days 3 and 4, and more so on day 4, as the route was well known and the driver did not pay as much attention to driving. According to [28], due to the rainy weather during the last two days of data collection, the driver’s state of fatigue may also have been accentuated by the use of windscreen wipers.

### 2.3. EEG and EOG Signal Analysis

**Figure 4:**
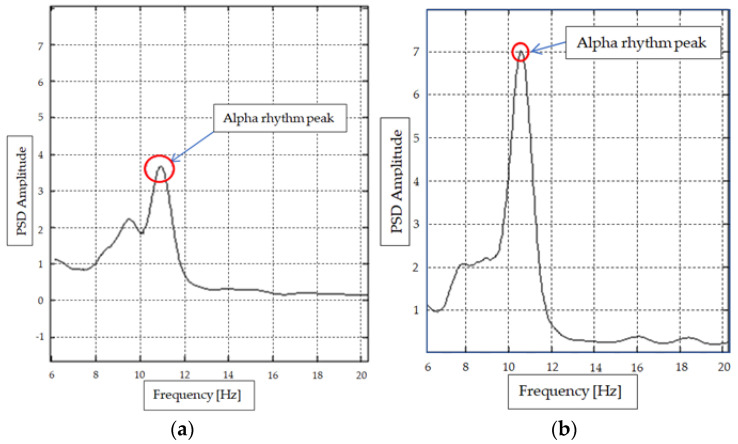
EEG signal diagrams, (PSD-power spectral density amplitude vs. frequency): (**a**)—driver in an alert state; (**b**)—driver in a drowsy state.

In [20], an application of EEG system for acquisition and data processing is presented. The aims of this research [20] are to analysis the signal peaks (of Alpha rhythm) in order to determine if the driver is in an alert state or a drowsy state, see Figure 4. Drowsiness is often associated with an increase in alpha brain waves. These are relatively low-frequency waves (8–12 Hz) that are dominant in the posterior regions of the brain when a person is awake but relaxed. As a person transitions from wakefulness to drowsiness, theta waves (4–7 Hz) might become more prominent. These are often associated with the early stages of sleep. Drowsiness can be characterized by an increase in slower frequencies, such as theta and delta waves, as the person moves closer to sleep.

Upon awakening, theta waves may still be present as the person transitions from sleep to wakefulness. Alpha waves might increase as the person becomes more alert. Beta waves (13–30 Hz) are associated with alert wakefulness. As the person becomes more awake and alert, beta activity in the EEG may increase. Delta waves (0.5–4 Hz), associated with deep sleep, should decrease as the person wakes up. It is important to note that these patterns may differ from person to person. The transition from sleep to wakefulness involves dynamic changes in EEG patterns.

**Figure 5:**
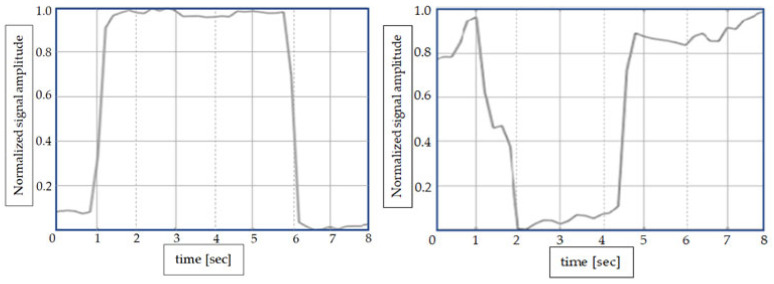
Types of signals recorded by EOG sensors (sensors’ positions are shown in Figure 2 EOG1, EOG2, EOG3) denoting S1 (**left**) and S2 (**right**).

Measuring EEG signals (Figure 4.) in drivers can be difficult, so if we have to choose between EEG and EOG signals, EOG signals are easier to obtain. The possibility of using EOG signals, collected by three sensors mounted in the eye area (Figure 2), was analyzed in other research [7,11]. In Figure 5, the two types of signal (S1, S2) are shown, which were identified after the preprocessing step. Combining these two types of signals let us distinguish between different movements of the eye and blinking. Recording these different types of eye movements for normal behavior and drowsy behavior can give us information about the state of the driver. The signals provided by the EOG1, EOG2, and EOG3 sensors (sensor positions are shown in Figure 2) regarding the driver’s eye blinking are as follows: S2 -> EOG1, S1 -> EOG2, S1 -> EOG3. The configuration of the 3 signals coming from the EOG sensors have unique configurations for each type of movement, so the configurations S2, S1, and S1 will not be encountered during any eye movement other than blinking. Typically, EOG1 and EOG2 are used to capture horizontal eye movements. The placement of electrodes allows for the measurement of electrical potential generated by the movement of the eyes from side to side. The positioning of the EOG3 electrode allows for the measurement of the electrical potential associated with up and down eye movement.

### 2.4. Face Image Analysis for Open or Closed Eye State Detection

For image classification of the driver state, a method using the neural network toolbox and the deep learning toolbox from MATLAB was studied [26,27]. The following premise was used: it assumes that drowsiness corresponds to the images where the driver’s eyes are closed and the alert state of the driver corresponds to the images where the eyes are open [29].

Two hundred images were used to analyze the drowsiness of the driver during the driving process. Half of these images are of the driver with open/half-open eyes, and the other half are pictures of the driver with their eyes closed.

**Figure 6:**
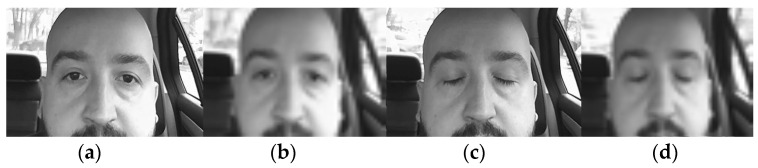
Images of the driver: (**a**)—with eyes open; (**c**)—with eyes closed. Images of the driver at low quality/resolution: (**b**)—with eyes open; (**d**)—with eyes closed [8].

In Figure 6, some images used for processing are shown. In order to minimize the huge amount of input data for the artificial neural network, the images were cropped and down-sampled; moreover, the processing time was diminished, and the overload of memory was also avoided.

In Figure 6b,d, images were down-sampled and sharpness was affected. This ensured a more robust recognition and classification. Furthermore, if the neural networks work well with low-quality images, it will surely work much better with high-quality images (Figure 6a,c).

### 2.5. Open or Closed Eye State Detection Using the EAR Algorithm

**Figure 7:**
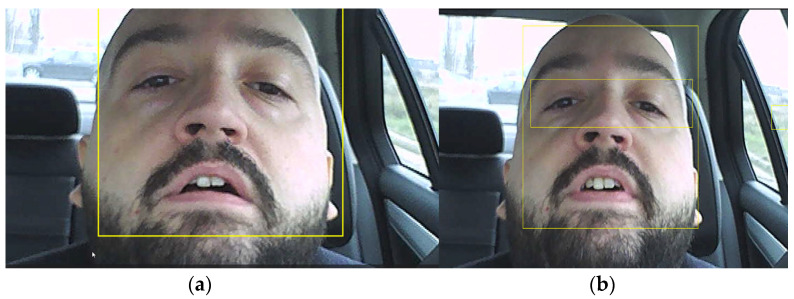
(**a**)—Face detection and tracking; (**b**)—Face and eye detection in streamed video.

The ratio between eye width and length uses a point recognition algorithm concerning face characteristics (point coordinates in pixels) and learned 68-point iBUG 300-W dataset features. Using the eye detection algorithm, the driver’s eyes were tracked as shown in Figure 7. In [29] the previous algorithm used in this case is described. The description is based on [30,31,32].

**Figure 8:**
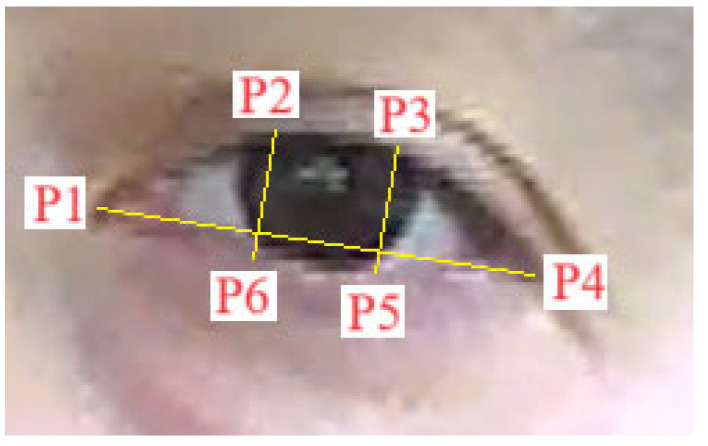
Eye aspect ratio (EAR) metric.

In Figure 8, the 6 characteristic points defined on the detected area of the eye are shown. Based on Relation (1), the EAR algorithm was computed and the results were split: high values mean the eyes were open, and low values mean the eyes were closed.

The Euclidian distance between the specified points are defined in Relation (1) with the double parting brackets [20].

(1) $$EAR=\frac{\left\|p_{2}-p_{6}\right\|+\left\|p_{3}-p_{5}\right\|}{2\left\|p_{1}-p_{4}\right\|}$$

where ‘*p_i_*’ (*i* = 1…6) is one of the points presented in Figure 8. Every point ‘*p_i_*’ has the coordinates given in pixels (*x_i_*, *y_i_*). The coordinates of the points are obtained from the image in Figure 7 by binarizing the image and then applying an edge detection algorithm (Sobel, Canny, Prewitt). The distance between the two points ‘*p_i_*’ and ‘*p_k_*’ is computed by the relation:

(2) $$\left\|p_{i}-p_{k}\right\|=\sqrt{{\left(x_{i}-x_{k}\right)}^{2}+{\left(y_{i}-y_{k}\right)}^{2}}$$

Relation (1) will be written as:

(3) $$EAR==\frac{\sqrt{{\left(x_{2}-x_{6}\right)}^{2}+{\left(y_{2}-y_{6}\right)}^{2}}+\sqrt{{\left(x_{3}-x_{5}\right)}^{2}+{\left(y_{3}-y_{5}\right)}^{2}}}{2\sqrt{{\left(x_{1}-x_{4}\right)}^{2}+{\left(y_{1}-y_{4}\right)}^{2}}}$$

### 2.6. Face Detection and Tracking Algorithms

**Figure 9:**
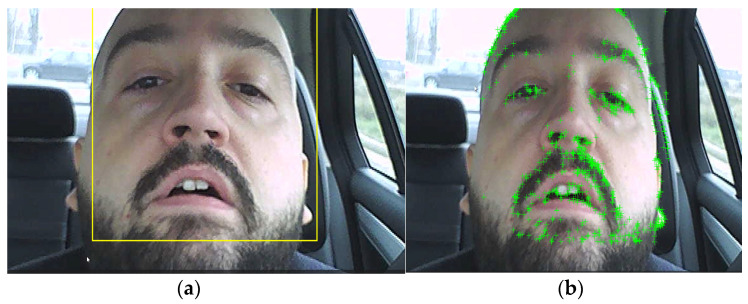
(**a**) Face detection using bounding box (yellow); (**b**) detected characteristic points (green +).

As an alternative to using the EAR algorithm trained on the iBUG 300-W dataset, another facial characteristic detection algorithm can be used, which has the potential to be more reliable. The algorithm is based on the Viola–Jones algorithm [33], which was developed to detect people’s faces, noses, eyes, mouth, or upper body. The algorithm is implemented in MATLAB as a cascade object detector which is trained on positive and negative images and relays on a series of successive classifiers. The detected bounding box is shown in Figure 9a. Once the area is reduced by detecting the bounding box, characteristic points are selected using a minimum eigenvalue algorithm developed in [34] to find feature points in a greylevel image. The algorithm is implemented in MATLAB using the ‘detectMinEigenFeatures’ function, and result is shown in Figure 9b.

Having detected the feature points, we can apply a point tracker algorithm, which in our case was the Kanade–Lucas–Tomasi (KLT) algorithm [35]. The point tracker algorithm is implemented in the MATLAB R2022b v9.13 language as a software object that follows a group of points using the feature-tracking method. This can be used for camera motion stabilization and estimation, and object following. The algorithm is satisfactory for tracking faces that are not out of plane or changing characteristics related to the image plane. The point tracker can be used in the majority of cases as a subroutine in a larger context where successive detections are needed.

**Figure 10:**
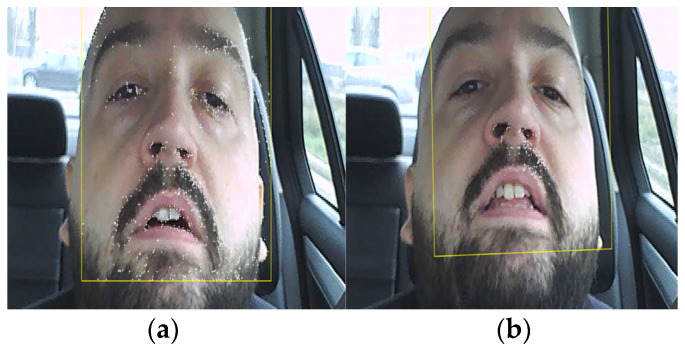
(**a**) Feature points before blinking; (**b**) loss of feature points after blinking.

**Figure 11:**
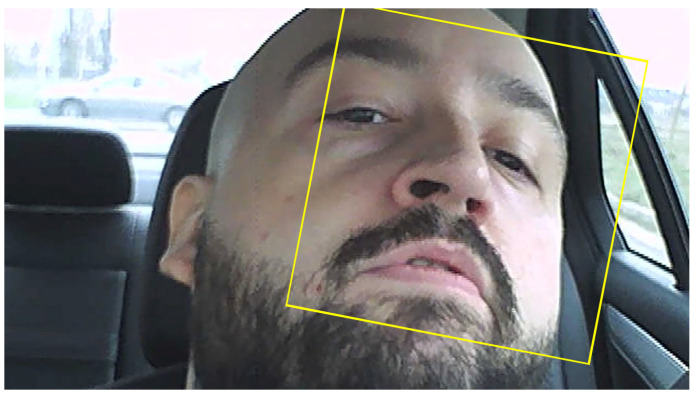
Tilted position of driver’s face followed by the bounding box and still detected by the algorithm.

Due to points that can be lost because of lighting changes or out of plane movement, to track a face over a longer time, successive acquisitions of feature points must be made. This can be observed in Figure 10, where in Figure 10a, in the area of the eyes, there are a large set of feature points which were lost after the driver blinked (Figure 10b). Even so, the algorithm is much more reliable than a variant which uses the eye detection variant (using the classification model ‘EyePairBig’ or ‘EyePairSmall’) when the detection is much worse. The face tracking algorithm in this case is sufficiently robust to work properly even in relatively extreme cases (Figure 11) [36,37,38].

**Figure 12:**
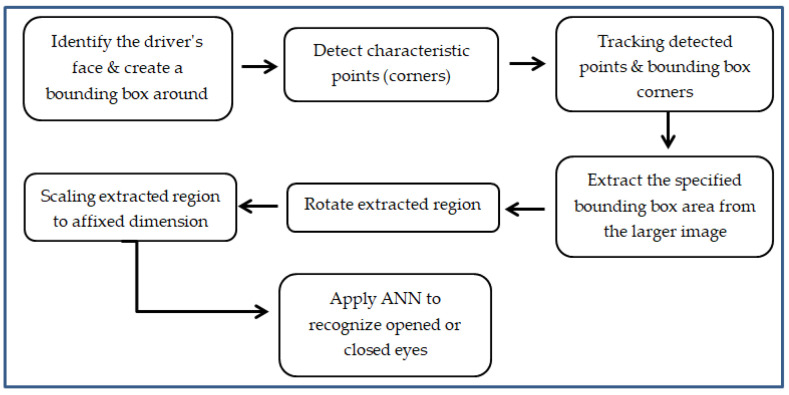
Block diagram of face detection and tracking algorithm.

Once the bounding box is firmly established, the following step is to find the timeline of the open or closed eyes. This step is similar to that described in the following Section 3.1. for other detection methods. Along with 1 hidden layer or an autoencoder ANN for eye state detection, we can also use a deep learning ANN, like, for example, a CNN (convolutional neural network) which nowadays are largely applied in image recognition. A flowchart for the described algorithms is shown in Figure 12.

### 2.7. The Use of Drowsiness Detection System in Autonomous Driving

Taking into account the classification based on SAE standards, it can be seen that only for the first 4 levels of autonomy is the driver’s intervention required, while for the last two levels of autonomy (level 4 and 5), the driver can be totally exempted from specific driving activities. Autonomous vehicles that can be encountered in traffic most often are those of maximum level 3; this is due to several reasons: technological limitations, legislative limitations, limitations in terms of infrastructure, etc. Thus, the implementation of the driver drowsiness monitoring system is beneficial to these categories of vehicles.

Since, up to level 3 of autonomy, the driver must be fit to drive or take over the controls when the system requires it, monitoring of the driver’s drowsiness must be carried out continuously, regardless of how the vehicle is used (autonomous mode or not). From this perspective, there are two different situations in which the detection system must work:

1. The vehicle is used conventionally, and the driver is in full control.

In this situation, if the system perceives that the driver is in a drowsy state, as a first measure, it will issue an acoustic warning and also a physical one through small vibrations of the devices that measure the biological signals, but also through vibrations in steering wheel. Next, there are two possibilities: the driver responds to the stimuli and tries to return to a state of heightened attention, or there is a situation where the driver does not respond to these stimuli (warnings). If the driver does not change his state following the warning signals, then through the ADAS system and based on the information provided by the fatigue monitoring system, the dynamic performance of the vehicle will be modified by reducing the speed and possibly by acting on the steering system if the level of autonomy allows this.

2. The vehicle (especially level 2 and 3) is operated with the autonomous driving function activated (the vehicle may have control over direction of travel, acceleration, and braking).

In the case of a level 3 autonomous car, even if the automatic driving system is in operation, the driver must be prepared to take control of the vehicle if the system requires this for various reasons (fails to correctly identify some obstacles due to weather conditions and thus fail to manage the situation). The driver must therefore be able to take over the controls. In such a scenario, the fatigue detection system can play an extremely important role as it detects the driver’s state and can warn them so that they do not fall into a state of deep drowsiness and can be ready to take over at any time. If driver fatigue is detected, the system will work in the same way as before.

## 3. Results

Regarding the results of EEG spectrum analysis, the decision of drowsy or alert state can be made either by detecting the peak amplitude in the 10–12 Hz domain (see Figure 4) or by applying a method as described in [19]. Both methods have been tested with fairly good results.

Regarding the results for the experiments using the EOG signals, the complete study is presented in [10]. Here, the results were very good with a good repeatability and consistency.

### 3.1. Results for the Face Image Analysis

The obtained results were processed using ANN and the fuzzy algorithm for the system to give a clear answer to the question of whether the state of drowsiness is reached or not. These algorithms are presented in the following sections.

#### 3.1.1. Artificial Neural Network with One Hidden Layer

**Figure 13:**
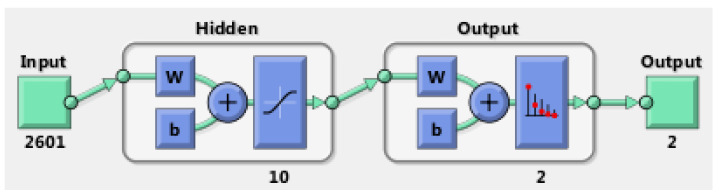
The structure of the network with 1 hidden layer [8].

A total of 70 images with eyes open or half-open as well as another 70 images with eyes closed were used to teach, validate, and also test the neural network. The total number of collected images was 200. The rest of the images (60 images) were kept for testing the working of the neural network after the training process was finished with the previous 140 images. The NN (neural network) was trained based on the structure presented in Figure 13. This structure was constructed based on 2601 neurons in the input layer, 10 neurons in the hidden layer, and 2 neurons in the output layer. Each neuron has its scope, so the neurons of the input layer match the number of elements of the input vectors (images) and the neurons in the output layer (two neurons) correspond to the possibilities of image classification. The inputs of the neural network are the images, which are treated as matrices and are read row by row. The input image for this case is Figure 6a, which is 416 × 416 in size, and Figure 6b, which has 2601 pixels (51 × 51 pixels), so the inputs for the network will be equal to 2601. There are two classes: drowsy/sleepy state or alert state [20].

In [9,39] the performance diagram and the error histogram are presented; both are obtained as a result of the neural network training process. From an analysis of the values from that performance training diagram and from the histogram boundaries, it can be said that there are some good results.

**Figure 14:**
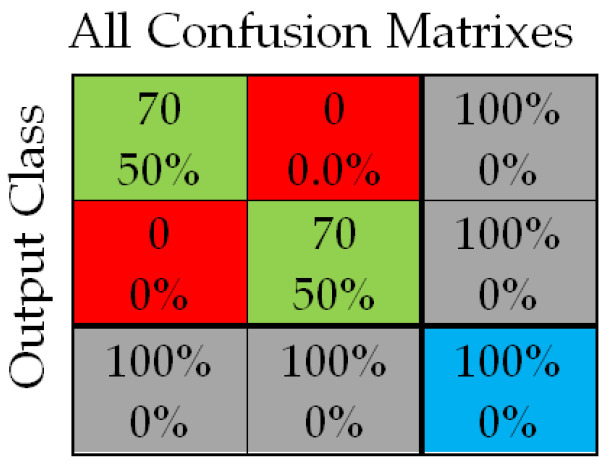
The training, validation, and testing matrix (confusion matrix) for one hidden layer network.

The Confusion matrix from Figure 14 shows the results of learning, validation, and testing. In such a matrix, the rows and the columns are used in the following way: a predicted class is represented by the each column and an actual class is presented by each row. The meaning of the colors in the matrix is as follows: green means a correctly classified sample and red means an incorrectly classified sample. In the case of the confusion matrix, the 3 × 3 configuration is due to the following fact: column 3 and row 3 of the matrix represent the sum of the two types of classified cases (wrongly classified and correctly classified). Analyzing the image, there can be seen that all have been correctly classified.

#### 3.1.2. Deep Learning Autoencoder Neural Networks

**Figure 15:**
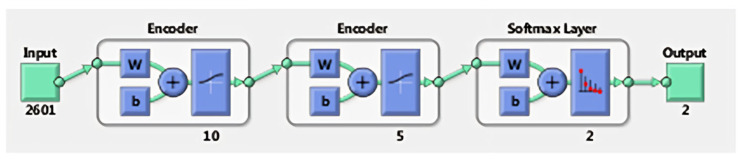
Network structure with 2 hidden layers [9].

For the autoencoder, the input and target vectors were similar, as in the case of the 1 hidden layer ANN. In the case of the autoencoder network, each layer was trained separately and then brought together in one network but with more layers, and the next step included the final training for the final entire network. The structure of this network is shown in Figure 15.

The training results of the autoencoder reveal promising outcomes. The training performance emphasizes the achieved minimum value, which signifies improved performance. It is evident that there were no occurrences of false positives or false negatives. This indicates precise classification across all tested images (30 with closed eyes and 30 with open/half-open eyes). In summary, these findings denote a satisfactory performance.

### 3.2. Results for the EAR (Eye Aspect Ratio) Algorithm

**Figure 16:**
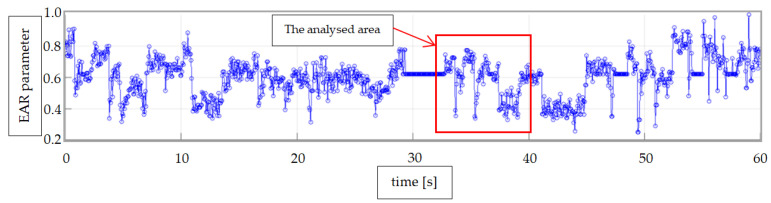
EAR algorithm results from video streaming.

**Figure 17:**
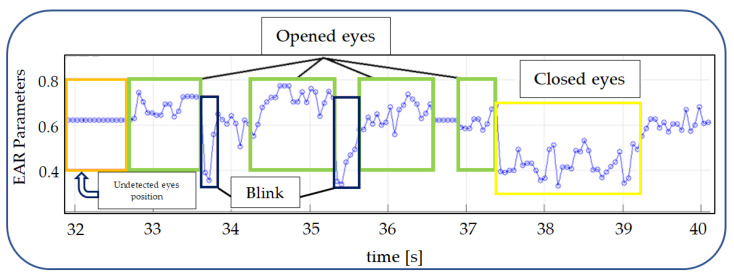
Detection of open or closed eye state by the EAR algorithm.

For the EAR algorithm, the same video was used (as for the ANN analysis), and that way, it was able to obtain a value of EAR for every frame, as presented in Figure 16. Also in Figure 16, an area of interest is marked, which will be analyzed in Figure 17.

In Figure 17, the area of the graph which corresponds to the different states of the eyes (closed or open) in the video is marked. Using a simple threshold method, the intervals can be more easily detected.

### 3.3. Results for the Face Image Analysis

**Figure 18:**
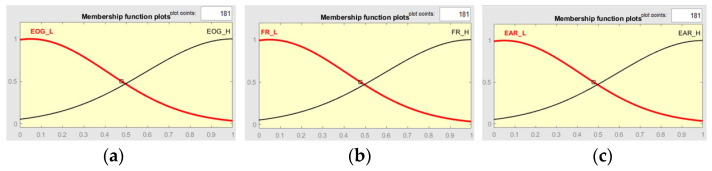
Input functions: (**a**)—EOG; (**b**)—FR (face recognition); (**c**)—EAR (eye aspect ratio); L = low value; H = high value.

In the case of image processing algorithms, there is a predisposition to errors due to the complexity of the input information (images). The detection accuracy could be seriously affected by many factors like: driver movements, environment influence, interior of the car, etc. In [8] was shown that the EEG signal detection, is influenced by the noise and sensor’s accuracy. In addition, it is difficult to use for driver drowsiness detection because it is uncomfortable. One of the methods to overcome the inconvenience and to improve accuracy is to use more systems in parallel, like the system presented above, and employ a decision system. One well-known multi-criteria decision method is the fuzzy algorithm, which can be used to create such a complex system. In this case, the input of the system is the result of the EEG/EOG processing together with the results of EAR image processing and face recognition. The input membership functions are shown in Figure 18.

**Figure 19:**
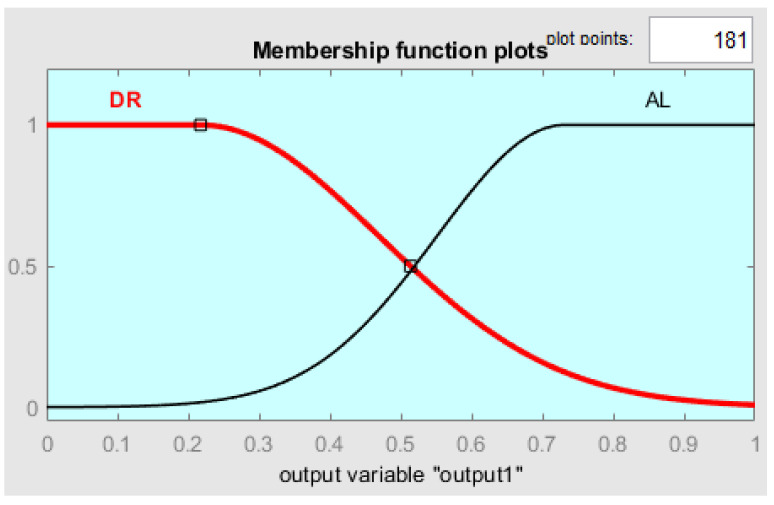
Output membership functions of the fuzzy system: DR—drowsy state; AL—alert state.

**Figure 20:**
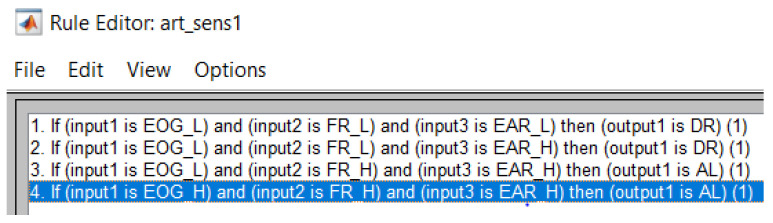
Rules that are applied in the system.

**Figure 21:**
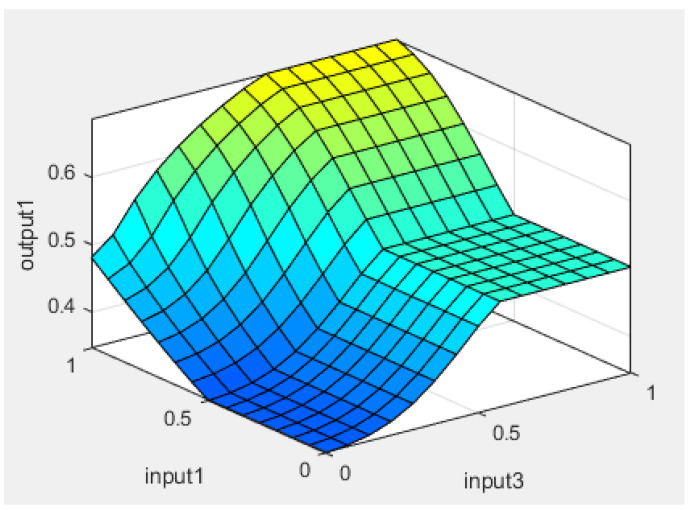
Decision surface for processing inputs [19].

Figure 19 presents the shape of the output membership function, using a Mamdani type output method. The system rule is defined thus: it is decided that the driver is in a drowsy state if at least two of the inputs are images with a closed eye state. The rules are presented more clearly in Figure 20 and Table 2. Figure 21 shows one of the decision surface models for processed inputs.

Since the decision of the detection system is based on a fuzzy algorithm, if we look at Figure 3, it is obvious that the three input elements provide information to the decision algorithm simultaneously. The first input element can be considered, the result of analyzing the patterns (signals) either from EEG in the case of Figure 3 or from EOG sensors, this depends on the method chosen. The other two inputs to be analyzed in the decision algorithm are the result of the facial recognition algorithm and the EAR algorithm or even the facial recognition and tracking algorithm in Section 2.6.

Table 2 shows a model of the rules applied by the fuzzy algorithm used to analyze the signals from at least 3 detection systems, in order to provide a clearer result on the drowsiness of the driver.

Considering recently published articles by other researchers in this field, we can see a high interest in this topic. For example, in [40,41], most of the methods that have been tested for determining driver fatigue are mentioned and presented. Another example of a system similar to the one presented in this article can be viewed in [42], where Igor et al. provide a more detailed analysis on the method of determining fatigue focusing on EEG signal processing.

## 4. Discussion

In this paper, a drowsiness detection system for drivers and its possibilities was presented. The system combines three types of signal (sensors): EEG, EOG, and image processing. The methods were described in more detail in previous works. Regarding the disadvantages of such systems, even though the results are good in laboratory settings, difficulties appear at the vehicle driver stage due to the reluctance of drivers to wear some electrodes (for EEG and/or EOG) on their bodies while driving. That is why the systems would be hard to implement in all cars in this configuration. Moreover, the biological parameters are different for every person, which means the system must be calibrated for every driver separately. There are some possibilities to increase the performance of every single system or make it more convenient for the driver to use it. Regarding the systems for monitoring the biological parameters (EEG, EOG), other types of electrodes (dry electrodes) could be implemented, which could be integrated into sunglasses, (fitness) bracelets, or other type of usual accessories, with wireless communication. In the case of image processing, both networks used and presented in previous section have a good results, but should be tested in more and different conditions to ensure a good accuracy and functionality in almost all possible condition.

This driver drowsiness monitoring system is similar to the perception, classification, and decision system used to identify the driving environment, based also on sensor fusion. Thus, for image processing, an algorithm similar to that for analyzing images taken by the front camera of the vehicle can be used to detect the position of the eyelids. The notable difference between the sign recognition algorithm and the drowsiness recognition algorithm is the database used to train and validate it.

Based on the results provided by the driver drowsiness detection system, control of the vehicle if the driver does not respond to warnings is achieved by means of the systems found in the standard vehicle construction specific to each level. Important changes to the autonomy system are in the decision and control algorithms.

## 5. Conclusions

As mentioned in the previous section, the system works well under laboratory conditions, and it is now necessary to develop a more convenient design solution for the driver. This system solution must have the property of self-calibrating according to the driver using the vehicle. The main purpose of such a system is to avoid accidents caused by drowsiness, when the ADAS is on or off. In order to be able to develop and implement such tools in the construction of the vehicle, legislation should be drawn up to encourage the use of such an equipment, even if it would only be used for professional drivers who usually drive for long periods of time. This would be an intermediate solution between the classic vehicle and the high-level autonomous vehicle (level 4 or 5 according with SAE standard). A brief analysis of the papers [43,44] shows that the systems needed to create an autonomous bus or an autonomous vehicle are extremely complex and numerous, involving technologies that still need to be validated before they can be said to be safe, but above all before they can take full control of the vehicle without the driver having to intervene in any way (this type of autonomy corresponds to the highest level of SAE classification, level 5).

To reduce the numbers of accidents due to the drowsy state of the driver and also to encourage this technology, the European Commission for Transport (European Parliament) elaborated a legislative framework for this problem. It gave the following regulation for the car manufacturers: ‘starting with 6 July 2022, all new model of vehicles should be equipped with a system for driver detection system, and starting with 7 July 2024, all new cars should have such a system’ [4,45].

In conclusion, until autonomous vehicles reach a stage of maturity, the driver monitoring and warning option, combined with other prevention/warning systems, would be a beneficial solution in terms of reducing the number of accidents with or without victims. The same is desired with the development of autonomous driving technology. Moreover, it is hoped that (autonomous) vehicles can be used by all people (including people with disabilities) to avoid traffic jams, etc. However, for all these stages, it is necessary to adapt the legislative framework in addition to developing the technology.

## Figures and Tables

**Table 1:** Information on the conduct of data acquisitions.

Day and Route No.	Total Sleep before Test	Route [km]	Driving (Recording) Time [min]	Weather	Time Interval	Comments
1	8 h	Oradea -> Turda -> Oradea (381 km)	332 min	cloudy	7:00 A.M.–2:00 P.M.	No/weak signs of fatigue
2	5 h 30 min	Oradea -> Arad -> Oradea (229 km)	200 min	cloudy/sunny	8:00 A.M.–11:45 A.M.	More frequent signs of fatigue
3	6 h	Oradea -> Carei -> Oradea (204 km)	175 min	rainy	11:00 A.M.–2:10 P.M.	Frequent signs of fatigue
4	7 h 30 min	Oradea -> Carei -> Oradea (204 km)	180 min	cloudy and rainy	11:00 A.M.–2:15 P.M.	Frequent signs of fatigue and lack of attention

**Table 2:** The decision rule for fuzzy algorithm.

EEG/EOG		Face Tracking-Recognition		EAR	Result
L	and	L	and	L	drowsy
L	and	L	and	H	drowsy
L	and	H	and	H	alert
H	and	H	and	H	alert

H = high(1); L = low(0); they represent the values of the measurement systems introduced in the decision algorithm.

## Data Availability

The data presented in this study are available on request from the corresponding author.
